# New Perspectives in Stroke Management: Old Issues and New Pathways

**DOI:** 10.3390/brainsci11060767

**Published:** 2021-06-09

**Authors:** Fabio Pilato, Rosalinda Calandrelli, Fioravante Capone, Michele Alessiani, Mario Ferrante, Gianmarco Iaccarino, Vincenzo Di Lazzaro

**Affiliations:** 1 Unit of Neurology, Neurophysiology, Neurobiology, Department of Medicine, University Campus Bio-Medico of Rome, via Álvaro del Portillo, 21, 00128 Rome, Italy; f.capone@unicampus.it (F.C.); m.alessiani@unicampus.it (M.A.); m.ferrante@unicampus.it (M.F.); gianmarco.iaccarino@unicampus.it (G.I.); v.dilazzaro@unicampus.it (V.D.L.); 2 Fondazione Policlinico Universitario Agostino Gemelli IRCCS, Roma-UOC Radiologia e Neuroradiologia, Polo Diagnostica Per Immagini, Radioterapia, Oncologia ed Ematologia, Area Diagnostica Per Immagini, 00168 Rome, Italy; rosalinda.calandrelli@policlinicogemelli.it

**Keywords:** stroke, stroke unit, head CT, CT angiography, thrombectomy

## Abstract

Stroke is a leading cause of disability and death worldwide and social burden is huge in terms of disabilities, mortality and healthcare costs. Recently, in an acute stroke setting, renewed interest in disease-modifying therapies and novel approaches has led to enhanced recovery and the reduction of long-term disabilities of patients who suffered a stroke. In the last few years, the basic principle “time is brain” was overcome and better results came through the implementation of novel neuroimaging tools in acute clinical practice, allowing one to extend acute treatments to patients who were previously excluded on the basis of only a temporal selection. Recent studies about thrombectomy have allowed the time window to be extended up to 24 h after symptoms onset using advanced neuroradiological tools, such as computer tomography perfusion (CTP) and magnetic resonance imaging (MRI) to select stroke patients. Moreover, a more effective acute management of stroke patients in dedicated wards (stroke units) and the use of new drugs for stroke prevention, such as novel oral anticoagulants (NOACs) for atrial fibrillation, have allowed for significant clinical improvements. In this editorial paper, we summarize the current knowledge about the main stroke-related advances and perspectives and their relevance in stroke care, highlighting recent developments in the definition, management, treatment, and prevention of acute and chronic complications of stroke. Then, we present some papers published in the Special Issue “Clinical Research on Ischemic Stroke: Novel Approaches in Acute and Chronic Phase”.

Stroke is a major cause of death and disability worldwide, and due to an increase in life expectancy, the increase of absolute and relative number of older people will make stroke an even more relevant issue, with a huge impact on healthcare and society. In the past decades, stroke management has been revised and major advances have been made for its treatment and prevention.

As healthy status includes maintaining good cognitive and physical functioning, avoiding or at least minimizing disease and disability, stroke represents a major challenge for health care and a main determinant to sustain the ability to stay healthy, not only in older age.

The biggest improvements in the stroke field were the awareness of the need to manage stroke patients in dedicated units (stroke units), in the acute phase, the development of reperfusion therapies and their implementations in clinical practice. Moreover, attention to some predisposing clinical conditions and risk factors allowed better clinical results, thereby reducing its incidence and improving prognosis.

After the first studies about systemic thrombolysis for acute stroke, now we are in an era of a steep increase in research about acute stroke care. Basic and clinical research allowed a fast-growing knowledge, changing our approach to cerebrovascular disease and, in particular, to acute stroke.

For acute treatment, the implementation of endovascular thrombectomy for large vessels occlusion to intravenous alteplase increased functional independence for more patients compared to thrombolysis, thus extending the time window for acute therapy. However, acute treatment was not the only field related to stroke management that has improved in the last few years. For example, the beneficial role of antiplatelet drugs in recurrent ischemic stroke prevention has emerged as greater than previously recognized. Additionally, other preventive strategies for recurrent stroke now include direct oral anticoagulants, as an alternative to warfarin in patients suffering from atrial fibrillation, and carotid stenting as an alternative to endarterectomy for symptomatic carotid stenosis.

Thanks to newly devised strategies for stroke management, stroke-related mortality rates are declining. However, we noted that prevalent stroke survivors, disability-adjusted life-years (DALYs) lost due to stroke and stroke-related deaths are growing and the global burden of stroke is increasing, causing huge direct and indirect social costs.

Novel advances in acute stroke management, along with a more comprehensive approach to primary and secondary prevention, improved stroke therapy. However, optimal stroke management requires targeting people at all levels of risk, which should be integrated with prevention strategies, also taking into account unhealthy lifestyles and risk factors shared with other diseases that predispose to cerebrovascular risk, such as diabetes and hypertension.

In recent years, research advances in basic sciences, radiological, clinical and therapeutic fields allowed the better management of stroke patients improving stroke diagnosis and therapy, extending time window and providing acute stroke therapies to patients who were excluded in previous trials.

At first, brain computer tomography (CT) was used only when making a differential diagnosis between ischemic and haemorrhagic stroke, but now it is used to assess large vessel occlusion and salvageable ischemic area as well, allowing a better selection of patients to enroll in specific acute treatments. These progresses improved the simple stroke concept of “time window”. Additionally, the clinical approach to stroke moved forward and more attention has been paid, not only to the acute treatment, but also to the management of the first days after stroke onset, and this has resulted in current guidelines recommending dedicated stroke units. These changes have allowed significant improvements in terms of outcome and recovery and, even if in-hospital mortality remains high for stroke, it has shown a marked decrease, mainly due to in-hospital complications avoidance. In fact, a more accurate management of feeding, blood pressure and glycemic control, in dedicated units during the acute period after stroke, allowed these clinical improvements.

The present collection of articles introduces some stroke-related research hot topics, in which significant improvements were recorded in recent years, and in particular, those in which we expect research to grow quickly in the near future.

In acute stroke patient assessment, the fast and accurate evaluation of large vessel occlusion, along with the salvageable ischemic area, was demonstrated to be the main point for effective acute stroke treatment, and they require advanced neuroimaging equipment and software tools, based on either CT or MRI.

Several tools have been proposed and this research field is rapidly growing. Verdolotti et al. [1] evaluated the effectiveness of a new semi-automatic post-processing software (ColorViz FastStroke, GE Healthcare, Milwaukee, WI, USA) in the evaluation of collateral circulation, compared to the commonly used six-point classifications of multiphase CTA. Two neuroradiologists separately evaluated 86 patients with anterior ischemic stroke symptoms who underwent multiphasic CTA and ColorViz. Their preliminary results showed that this tool may have a diagnostic performance comparable to CTA. In addition to this, it is capable of reducing the evaluation time, allowing the fast assessment of collateral vessels.

Neuroradiological tools in acute stroke evaluation are even more important because they allow a faster differential diagnosis between small vessels and large vessel occlusion (LVO) and enrolment in acute treatments schemes and, in particular, mechanical thrombectomy.

In recent years, mechanical thrombectomy has become the standard therapy in patients with acute ischemic stroke (AIS) due to LVO, but several techniques may be used alone or in combination. The main purpose of these techniques is to achieve fast and complete vessel recanalization avoid complications. These techniques involve direct aspiration and stent retriever, but their choice mainly depends on both operator preference and anatomical evaluation. However, the best choice is difficult because head-to-head results’ comparisons may be challenging. Several studies have tried to solve this clinical issue, but a solution is still to be found. In a retrospective study, involving 76 patients, the procedural efficacy of the direct aspiration technique, using Penumbra ACE^TM^ aspiration catheter and the stent retriever technique with a Solitaire^TM^ FR stent, was compared [2]. Results showed comparable clinical outcomes, suggesting that both techniques may be used, possibly in different contexts.

Clinical research about acute stroke reperfusion therapies has always been focused mainly on anterior circulation stroke, even though posterior strokes may be a devastating disease too.

Anterior circulation has always been a main topic in acute stroke for both thrombolysis and thrombectomy. For this reason, the most common stroke-related scales, currently used in clinical practice, are used for anterior circulation and they are either of limited value or not informative at all for posterior circulation. Nevertheless, posterior circulation strokes account for 20–40% of all ischemic strokes. Some studies reported usefulness of treating stroke in posterior circulation, and probably, in the near future, stroke treatment will also be implemented for posterior stroke, but specific clinical scales are lacking. Wisniewski et al. [3] proposed a new seven-item dedicated scale called Adam’s Scale of Posterior Stroke (ASPOS) to exclusively assess the severity of posterior circulation strokes. They designed a prospective observational study, involving 126 posterior circulation ischemic stroke subjects. Four researchers, previously trained in ASPOS, randomized the stroke severity using ASPOS tool and other appropriate stroke scales (The National Institute of Health Stroke Scale—NIHSS, modified Rankin Scale—mRS, Glasgow Coma Scale—GCS, Barthel Index-BI, or Israeli Vertebrobasilar Stroke Scale—IVBSS) to assess the reliability and validity of ASPOS and investigate its predictive value. They reported that ASPOS is a valid and reliable tool, and that it can be used to more accurately select candidates for specific treatments, and it may also have additional predictive properties.

There is growing interest in the use of new biomarkers capable of predicting stroke outcomes, but their use in clinical practice is quite limited. Kim et al. [4] explored the impact of glycated albumin (GA) on short-term functional outcomes, as measured using the modified Rankin Scale (mRS) at 3 months, compared to glycated hemoglobin (HbA1c) in 1163 AIS patients from two hospitals. After adjusting for multiple covariates, the higher GA group (GA ≥ 16%) had a 1.4-fold risk of having unfavorable mRS, suggesting that GA level might be a novel prognostic biomarker compared to HbA1c for short-term stroke outcome.

Another hot topic in stroke research is the potential hemorrhagic transformation (HT) of ischemic stroke and in particular symptomatic hemorrhagic transformation (sHT). Hemorrhagic transformation is a complication of AIS and it may be induced or favoured by reperfusion therapies, but it can also be part of the natural course of ischemic lesions. Symptomatic hemorrhagic transformation (sHT) is a life-threatening complication of acute ischemic stroke (AIS). The early identification of patients at an increased risk of sHT can have clinically relevant implications, but a marker linked to sHT is still lacking. As AIS elicits a robust activation of the immune system and even more HT, Świtońska et al. explored the association of neutrophil-to-lymphocyte ratio (NLR) at admission with sHT and they found that NLR can predict sHT in patients with AIS undergoing revascularization [5].

Current guidelines recommend that stroke patients should be managed in dedicated stroke units. However, thrombectomy has added new issues due to the intensive care needed by some patients before and after the procedure. Management in an intensive care unit may be an additional risk factor for stroke patients and it is associated with poor prognosis [6]. In a prospective cohort study, 158 AIS patients admitted to a neuro-intensive care unit (NICU) after thrombectomy were studied, in order to assess factors linked to functional outcomes defined as independency (mRS < 2). IVT and nasogastric tube removal was associated with better outcomes at 3 months, whereas older age and hemorrhagic transformation were linked to 6-month independency, suggesting that acute stroke patients with LVO who require NICU management soon after IAMT may show specific clinical factors that influence short- and long-term prognosis [7]. These results suggest that short-term outcomes may be linked to feeding and probably dysphagia. Dysphagia is a relevant topic in acute stroke management. In fact, AIS patients are known to manifest a decreased cough force, which is associated with an increased risk of aspiration, and lesions of specific brain regions have been linked to impaired reflexive coughing. Bo Lee et al. [8] evaluated this topic, in a retrospective MRI study. They found that when AIS involved some brain regions such as the sub-gyral frontal lobe, the superior longitudinal gyrus and the posterior corona radiata, patients demonstrated that they had a weak cough flow; moreover, lesions in the inferior parietal and temporal lobes and both the superior and mid-temporal gyrus were associated with a weak peak cough flow during voluntary coughing. The results of this study might be useful in predicting, and probably better assessing, patients who are at risk of poor cough function and thus at risk of aspiration pneumonia.

Another important issue, far from being resolved, is the blood pressure management in acute stroke patients that became even more complicated after the advent of IAMT. In fact, despite the large amount of data on the preventive strategies and therapeutic measures that can be adopted, the management of high BP in patients with acute cerebrovascular diseases presenting at the emergency department is still an area of debate. Cantone et al. [9] provide a timely updated review on the current treatment, debated issues, and future directions related to hypertensive crisis in patients who are having a cerebrovascular accident. They also focus on the management of stroke-related, time-dependent interventions, such as intravenous thrombolysis and mechanic thrombectomy.

In recent years, a great amount of progress has been made in the field of antithrombotic therapies. After the introduction of clopidogrel as an alternative antiplatelet therapy for atherothrombotic stroke, new oral anticoagulants have been introduced for cardioembolic stroke due to atrial fibrillation. However, some concerns have emerged about clopidogrel resistance. Previous studies have revealed that, in acute stroke patients, high platelet reactivity in patients treated with clopidogrel may worsen their prognosis. In a prospective, single-center observational study, enrolling 74 AIS patients, Wisniewski et al. [10] highlighted that the dynamics of platelet reactivity over time predict the clinical course and prognosis of stroke better than a single value.

When it comes to the risk factors, non-valvular atrial fibrillation (NVAF) is the most common cause of cardioembolic stroke, which is the most severe ischemic stroke subtype. Even if some improvements were made in preventive antithrombotic therapies after NOAC introduction, few steps forward were made in acute antithrombotic therapies and there is no agreement about when it is appropriate to start or restart therapy with anticoagulant drugs. In an observational prospective uncontrolled study [11], which enrolled 75 elderly AIS patients with moderate disability, Edoxaban was introduced during acute stroke. They did not observe any symptomatic intracranial bleeding or recurrent stroke after 3 months of treatment with early administration of Edoxaban, while two gastrointestinal major bleedings, and 11 minor bleedings were reported. Even if these results should be confirmed in a larger trial, due to the small sample size, they suggest that Edoxaban, used early after acute stroke, seems to be safe in some patients after cardioembolic stroke.

Finally, the role of vascular risk factors in determining long term consequences of stroke such as cognitive impairment is still a point of debate. Post-stroke cognitive impairment (PSCI) is one of the major disabilities encountered by post-stroke survivors. As a potential risk factor for PSCI, multiple glucose parameters such as hemoglobin A1C, hyperglycemia, glycemic variability, and glucose dynamics have received significant attention for their implication in both diabetic and non-diabetic patients. As post-stroke hyperglycemia is a frequent finding in acute ischemic stroke patients, and due to its association with poor functional and cognitive outcomes, Lee et al. [12] investigated this issue in an observational retrospective study, which assessed the association between the glycemic gap on admission and PSCI in ischemic stroke patients. They demonstrated that an elevated glycemic gap is significantly associated with PSCI three months after a stroke, with preferential involvement of frontal and memory domain dysfunctions, concluding that hyperglycemia should be considered, along with glycemic control status at the time of stroke onset for the prediction of cognitive outcome after ischemic stroke.

There are still many unexplored topics in the acute and post-stroke management field, but in this Special Issue, we focused on the most promising research fields that hopefully, in the next few years, will develop significant clinical changes in stroke patients management.

In conclusion, we hope that the studies shown in this Special Issue will stimulate interest for further basic and clinical investigations on cerebrovascular diseases, promoting the research for a better acute management that will enhance stroke recovery, reducing patients’ disability and social burden.
